# The Unusual Invader in a Patient with Long-Standing Rheumatoid Arthritis: A Case of *Leishmania major* Colonization of Rheumatoid Nodules

**DOI:** 10.3390/dermatopathology13010008

**Published:** 2026-01-27

**Authors:** Monia Di Prete, Viviana Lora, Arianna Lamberti, Alessandra Latini, Carlo Cota

**Affiliations:** 1 Dermatopathology Research Unit, San Gallicano Dermatological Institute IRCCS, Via Elio Chianesi, 53, 00144 Rome, Italy; monia.diprete@ifo.it (M.D.P.);; 2 Dermatology, San Gallicano Dermatological Institute IRCCS, Via Elio Chianesi, 53, 00144 Rome, Italy; 3 Dermatology, Department of Medical, Surgical and Neurological Science, University of Siena, 53100 Siena, Italy; 4 STI/HIV Unit, San Gallicano Dermatological Institute IRCCS, Via Elio Chianesi, 53, 00144 Rome, Italy

**Keywords:** rheumatoid arthritis, rheumatoid nodule, cutaneous leishmaniasis, dermatopathology, immunosuppression

## Abstract

Rheumatoid nodules are the most common extra-articular manifestation of rheumatoid arthritis. Long-term immunomodulatory therapies, including corticosteroids, used in the management of rheumatoid arthritis are associated with a higher risk of infections. Leishmaniasis is a neglected protozoal infection that may arise in these patients. Cutaneous presentation is the most common and is characterized by a wide spectrum of clinical manifestations and courses, depending on the interplay between species involved and the host’s immune response. Here, we report the rare and intriguing case of a patient with long-standing rheumatoid arthritis, chronically treated with systemic prednisone, whose rheumatoid nodules were colonized by *Leishmania major.* In this context, therapeutic strategies must be tailored to species and patient factors. This report expands the differential diagnosis of rheumatoid nodule, highlighting the importance of considering opportunistic infections in exuberant presentations, particularly in immunosuppressed patients coming from or travelling in endemic regions. Intracellular pathogens may exploit the localized immunological niche represented by the rheumatoid nodule of an immunocompromised host to survive and replicate undisturbed. It also underscores the value of the clinico-pathological correlation and the importance of integrating molecular analyses to identify unexpected microorganisms that can be hidden by concomitant disease, avoiding misdiagnosis, ensuring timely treatment, and improving patients outcomes.

## Simple Summary

Rheumatoid arthritis is an autoimmune disease characterized by synovitis, joint destruction, and extra-articular manifestations, such as rheumatoid nodules. Its management relies on immunomodulatory therapies, including corticosteroids, which may cause immunosuppression and, consequently, infections. Leishmaniasis is a zoonosis caused by intracellular protozoa, transmitted through sandfly bites, and cutaneous leishmaniasis is the most common presentation. In immunocompromised patients, the parasites may disseminate, resulting in atypical manifestations. This case is notable as the patient developed diffuse cutaneous leishmaniasis due to her impaired cell-mediated immunity, with microorganisms colonizing pre-existing rheumatoid nodules. Unlike previous observations, this was a primary cutaneous infection rather than a reactivation, suggesting that *Leishmania* may exploit localized immunologically impaired niches, such as the rheumatoid nodule, to survive and, possibly, disseminate. Molecular techniques improve diagnostic accuracy and characterization of leishmaniasis, supporting histopathology and guiding appropriate treatments, confirming the synergistic approach combining conventional microscopy and molecular biology that should be adopted.

## 1. Introduction

Rheumatoid arthritis (RA) is a chronic systemic autoimmune disease characterized by persistent synovial inflammation, progressive joint destruction, and extra-articular manifestations, leading to progressive disability, significant morbidity, and increased mortality [1]. Among extra-articular localizations, rheumatoid nodules (RNs) are the most common cutaneous manifestation, occurring in up to 40% of patients, particularly those with long-standing, seropositive disease [2]. RNs typically develop at the site of repeated mechanical stress, which may be represented by minor trauma, and are microscopically characterized by palisading necrobiotic granulomas with a trizonal architectural pattern: a central area of fibrinoid necrosis, surrounded by activated macrophages and, finally, a peripheral lymphoplasmacytic infiltrate [2].

Therapeutic strategies to manage RA rely on disease-modifying anti-rheumatic drugs and immunomodulatory agents, including corticosteroids, which remain widely used for their fast and strong anti-inflammatory action. However, the latter induces generally chronic immunosuppression, which is associated with a higher risk of infectious complications [3], including those caused by opportunistic and atypical pathogens. In this setting, the diagnosis may become particularly challenging for both dermatologists and dermatopathologists, as the cutaneous manifestations may be characterized by unusual clinical presentation and histopathological features.

Leishmaniasis is a neglected zoonotic protozoal infection caused by various species of the obligate intracellular, flagellated parasites of the genus *Leishmania*, transmitted to humans and other mammals through the bite of infected phlebotomine sandflies. The disease represents a major global public health concern, affecting an estimated 12 million people worldwide. Cutaneous leishmaniasis (CL) is the most common form of the disease and may present with a wide spectrum of clinical manifestations and courses, ranging from localized and self-limiting lesions to disseminated forms, depending on the interplay between the infecting species and the host’s immune response [4]. In the Old World, *L. major* is one of the most frequent causes of CL, particularly in the endemic regions of North Africa and the Middle East [5].

Although CL is typically confined to the skin, in immunocompromised patients, the parasites may disseminate, resulting in multiple and atypical lesions, mimicking other infectious, inflammatory, or even neoplastic conditions. The potential of the parasites to spread in the organism highlights the role of both local and systemic host’s immune competence in the disease pathogenesis and progression. The occurrence of leishmaniasis within pre-existing lesions of other inflammatory conditions is exceedingly rare and not yet clearly understood. In particular, the colonization of RNs by *Leishmania* spp. has been described only in a handful of cases [6,7,8], and its pathogenetic mechanisms remain unclear. The granulomatous microenvironment of RNs, characterized by an impaired local immune surveillance, may represent a localized immunological niche, raising the hypothesis that *L. major* may exploit this context to escape the immune system response and proliferate.

Herein, we report the rare and intriguing case of a patient affected by long-standing RA treated with chronic corticosteroid therapy, in whom RNs were found to be colonized by *L. major*. This case shows an unusual clinico-pathological association, underscoring the potential clinical and histopathological pitfalls, and highlights the importance of molecular analysis integration in the diagnostic algorithm of infections, with the aim of avoiding misdiagnosis and rapidly establishing the most effective treatment in immunosuppressed patients manifesting atypical cutaneous lesions.

## 2. Detailed Case Description

A 59-year-old woman originally from Morocco was referred to the Dermatology Unit of our institute for a two-month history of progressive skin lesions involving the face and extremities. Her daughter, who was already residing in our country, arranged her transfer from the original country for diagnostic assessment and consequent treatment. Her past medical history included long-standing RA, undifferentiated connective tissue disease, pulmonary fibrosis, osteoporosis, and chronic normocytic anemia. She had been receiving chronic systemic treatment with prednisone (at variable doses over several years) in association with hydroxychloroquine. No recent changes in her home therapy nor history of biologic agent administration were reported.

**Figure 1:**
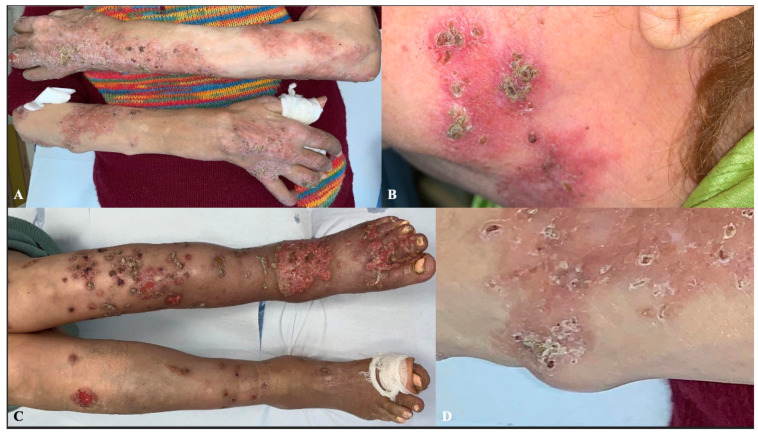
Clinical presentation. Multiple erythematous and scaly plaques, predominantly located on the extremities (**A**,**C**) and face (**B**). One of the lesions evolved into subcutaneous nodules, which was biopsied (**D**).

At the physical examination, there were multiple erythematous and desquamative patches and plaques, covered by crusts, predominantly located on the upper and lower limbs and, in a minority, on the head-neck district. Some lesions had evolved into non-tender, subcutaneous nodules. Moreover, the patient presented with slightly painful papules and nodules on the limbs, many of which were ulcerated and coalesced into large ulcers on the dorsal aspect of the feet (Figure 1). According to the history of long-standing RA, some of the nodules were consistent with RNs, based on their location on pressure points and clinical morphology, but the crusted lesions looked unusual. No fever, weight loss, or systemic symptoms, other than those associated with her known comorbidities, were reported by the patient. No lymphadenopathies were particularly suspicious. Laboratory investigations, including complete blood count, liver and renal functionality, and inflammatory markers, were unremarkable.

**Figure 2:**
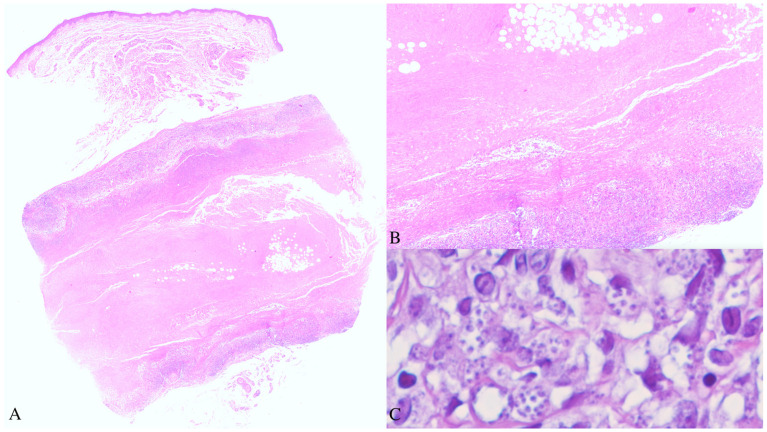
Histopathological aspects. (**A**) Well-demarcated focus of fibrinoid necrosis in the deep dermis-subcutis, surrounded by palisading histiocytes and a peripheral mixed inflammatory infiltrate (Haematoxylin-eosin stain. Original magnification: 20×). (**B**) A particular showing the palisading histiocytic inflammatory infiltrate surrounding dense fibrinoid necrosis (Haematoxylin-eosin stain. Original magnification: 100×). (**C**) At higher magnification, numerous small, round, pale-blue bodies consistent with the amastigote form of *Leishmania* spp. were observed within the cytoplasm of the histiocytes (Haematoxylin-eosin stain. Original magnification: 400×).

As the clinical aspects were not completely clear and given the patient’s immunosuppressed status, a broad differential diagnosis was considered, including both infective and inflammatory diseases. A skin biopsy was obtained from a subcutaneous nodule of the right forearm. The histopathological examination revealed a well-demarcated focus of fibrinoid necrosis located in the deep dermis, surrounded by palisading histiocytes and a peripheral mixed inflammatory infiltrate composed predominantly of lymphocytes and plasma cells, consistent with the diagnosis of an RN. At higher magnification, numerous small, round to oval, pale-blue bodies, measuring 2–4 microns in maximum diameter, were observed within the cytoplasm of the histiocytes and scattered among inflammatory cells. Their morphology was consistent with the amastigote form of *Leishmania* spp. (Figure 2). Even though the patient had lived for most of her life in an endemic country for leishmaniasis, she confirmed not being diagnosed with this disease ever before.

Given the unexpected histopathological finding, further diagnostic investigations were performed. Real-time polymerase chain reaction (PCR) analysis on the same paraffin-embedded formalin-fixed sample used for the microscopic examination confirmed the presence of *L. major* DNA. Therefore, according to clinical, histopathological, and molecular findings, the diagnosis of CL due to *L. major* colonizing a pre-existing RN was established.

The patient was subsequently treated with intravenous liposomal amphotericin B at a daily dose of 3 mg/kg for 10 consecutive days. The therapy was well-tolerated, without significant adverse effects. An evident clinical improvement was observed, with the progressive healing of the crusted lesions and the reduction in the size of the more infiltrated plaques and nodules. At the follow-up examination, no evidence of the infectious disease was noted.

## 3. Discussion

Leishmaniasis is a vector-borne zoonosis caused by obligate intracellular protozoa of the genus *Leishmania*, transmitted to humans and other mammals through the bite of infected phlebotomine sandflies (*Phlebotomus* species in the Old World and *Lutzomyia* species in the New World). It affects approximately 12 million people worldwide, with an estimated incidence of 0.7–1 million new cases occurring annually. Despite being considered a neglected tropical disease, leishmaniasis remains a significant global public health problem, particularly due to the absence of an effective vaccine and the limited availability of safe and affordable treatments [4,9,10,11]. Moreover, it represents a significant diagnostic and therapeutic challenge, mainly in immunocompromised patients, who may present atypical clinical and histopathological manifestations.

In the Old World, the most commonly transmitted species include *L. donovani*, *L. infantum*, *L. major*, *L. tropica,* and *L. aethiopica* [10,11]. The clinical spectrum of leishmaniasis is protean, ranging from purely cutaneous involvement (CL), with localized and self-healing ulcers, to more severe and diffuse cutaneous (DCL), muco-cutaneous (MCL), and visceral forms (VL), in which non-ulcerative plaques and nodules may occur. This heterogeneity depends on the complex interaction between the infecting parasite species and burden and the host’s immune system response. Consequently, in immunocompetent people, CL is the most common clinical presentation and is endemic in more than 90 countries across tropical and subtropical regions, while immunosuppressed patients—including those with autoimmune diseases and those receiving long-term immunomodulatory treatments—generally experience more aggressive and generalized forms. The cutaneous involvement by leishmaniasis typically manifests as one or several papules or nodules that may ulcerate and heal, evolving into disfiguring scars. However, from a clinical point of view, many atypical lesions have been described in the course of this disease, including panniculitic-like, lupoid, psoriasiform, sporotrichoid, impetigoid, erysipeloid, papular, verrucous, chancriform, hemorrhagic, and ulcero-crusted morphologies, mimicking inflammatory, infectious, or neoplastic diseases, respectively. These lesions are often asymptomatic but can become painful when located near joints or if secondarily infected.

From a histopathological point of view, CL may present with a spectrum of patterns, reflecting the stage of the disease and different degrees of host immune competence, ranging from a dense polymorphic dermal inflammatory infiltrate in the early lesions—composed of lymphocytes, plasma cells, and numerous parasitized macrophages—to granulomatous inflammation with few or absent amastigotes in the later stages [12]. In the latter scenario, the identification of *Leishmania* amastigotes within the granuloma may be challenging but remains the diagnostic gold standard. In immunocompromised patients, the impaired inflammatory response may result in a high parasite burden and exuberant clinical and histopathological features. In our case, the overlapping prototypical palisading necrobiotic granuloma of the RN may have represented a diagnostic pitfall, as its architecture could have overshadowed the underlying parasitic infection, which could have been overlooked at the scanning magnification evaluation and misinterpreted as part of the inflammatory process. Therefore, a high index of suspicion and careful histopathological examination at high power are crucial to identify amastigotes within the cytoplasm of the histiocytes composing the inflammatory infiltrate, especially in immunocompromised patients coming from or travelling to endemic countries. The histopathological differential diagnoses of leishmaniasis include other mycotic and parasitic infections, particularly histoplasmosis, caused by *Histoplasma capsulatum*, a dimorphic saprophytic fungus closely resembling *Leishmania* in size and shape on tissue sections from formalin-fixed, paraffin-embedded samples. However, the two organisms present different morphology at the microbial culture and travel history. Histochemical stains, such as periodic acid-Schiff or Grocott methenamine silver, may help in differentiating these two microorganisms, as Leishmania is a protozoan parasite and does not stain with these techniques, whereas Histoplasma is a fungus and is positive for both stains. Nowadays, there are no ancillary specific staining to detect *Leishmania* on histopathological sections. Giemsa may facilitate the identification of the microorganism, but Haematoxylin-eosin is still the gold standard.

The advent of molecular diagnostic techniques, particularly real-time PCR, significantly improved the diagnostic accuracy and characterization of leishmanial infections, allowing species identification with both sensitivity and specificity rates exceeding 90% [12,13]. In our case, the molecular confirmation of *L. major* was essential to support the microscopic findings and to guide the appropriate treatment approach, confirming the synergic role between conventional microscopy and molecular techniques.

The pathogenesis of leishmaniasis depends on the complex interplay between the host’s immune system competence and parasitic virulence, determining the self-resolution or the chronic course of the lesions. A balanced Th1/Th2 response is critical for the lesion’s outcome: a predominant Th1-mediated response leads to effective parasite control and disease resolution, while a Th2-skewed inflammation is associated with persistent infection. In fact, previous studies suggested that parasite dissemination in DCL may be due to the impairment of the peripheral Th1 cell-mediated immunity [9,14].

Despite advances in RA management, corticosteroids remain a cornerstone in the therapeutic algorithm due to their strong anti-inflammatory action. However, their prolonged use is also associated with an increased risk of infectious complications, especially when administered at high doses. Although clinical guidelines recommend short-term use, long-term corticosteroid therapy remains a common practice, with estimates ranging from 30% to 60% among RA patients [3,15]. Previous evidence indicated that corticosteroids inhibit Th1 immunity, decreasing the production of interleukin (IL)-12, interferon (IFN)-gamma, IFN-alpha, and tumour necrosis factor (TNF)-alpha by antigen-presenting cells. On the other hand, they upregulate IL-4, IL-10, and IL-13, releasing a Th2 response. Through these mechanisms, the chronic administration of systemic corticosteroids is responsible for a selective suppression of the Th1-axis and a shift toward Th2-mediated humoral immunity, rather than a generalized immunosuppression [16]. In our patient, the chronic and selective immune dysregulation of the Th1 compartment due to the long-term corticosteroid therapy compromised the host defences, predisposing to intracellular infections and eliciting the atypical and severe presentation of DCL observed in this case.

RNs are the most common extra-articular manifestation of RA, particularly in patients with seropositive disease, occurring in up to 35–40% of cases. They typically develop over pressure points or repeated minor trauma sites and are represented by non-tender, subcutaneous swellings [17,18]. Histopathologically, RNs are characterized by palisading necrobiotic granulomas, in which three zones can be classically recognized: a central area of fibrinoid necrosis, surrounded by palisading histiocytes, and a peripheral mixed inflammatory infiltrate, composed of lymphocytes and plasma cells [2,18]. The histopathological differential diagnoses of RN are particularly broad and include conditions characterized by granulomas surrounding a variable amount of degenerated material. Granuloma annulare is an idiopathic, self-limited dermatosis not associated with concomitant joint disease, presenting palisaded granulomas with minimal necrobiosis and variable interstitial mucin deposits. Necrobiosis lipoidica is a dermo-hypodermitis of the anterior aspect of the legs developing more frequently in diabetic patients with the distinguishing histopathological sign of the “layered cake”, due to the alternance of large foci of necrobiosis and lymphoplasmacytic and histiocytic inflammatory infiltrate involving the full thickness of the dermis, with possible spreading into the adipose tissue septa. Palisaded neutrophilic granulomatous dermatitis is generally the expression of an underlying condition, such as autoimmune connectivitis (also RA), lymphoproliferative disorders, or infections, and is represented by palisaded granulomas entrapping single collagen fibres accompanied by neutrophilic remnants. Gout tophi are the cutaneous manifestation of an underlying chronic gout arthritis, most commonly affecting the great toe metatarsophalangeal joint and associated with hyperuricemia, microscopically constituted by needle-shaped aggregates of urate crystals that dissolve during the processing and appear as clear oval spaces surrounded by foreign body giant cell reaction. Finally, calcinosis cutis is represented by well-circumscribed dystrophic calcifications surrounded by the host’s giant cell reaction at the site of previous minor trauma or inflammation.

The present case is notable for several reasons. First, the patient developed multiple lesions involving non-contiguous anatomical regions consistent with DCL, a rare and severe form usually associated with impaired cell-mediated immunity, likely related in this case to the long-standing RA treated with prolonged administration of systemic corticosteroids, a well-known predisposing factor to opportunistic infections. Then, and most intriguingly, *L. major* was isolated within a pre-existing RN, an unusual and rarely reported finding that remains poorly understood from a pathogenetic perspective. We speculated that this finding represents a collision of the two diseases of the patient, as the newly discovered CL—which, to our knowledge, is not typically associated with necrobiotic granulomas—colonizes the RNs, expressing the well-documented chronic immunosuppressive disease affecting this woman. Finally, this case is a rare occurrence. Unlike other cases described in the literature [6,7,8], it did not represent the relapse or reactivation of a previous infection, which seems to be the most frequent pathogenetic mechanism underlying this curious association, but with a primary cutaneous form. This observation raises the hypothesis that *L. major* intracellular amastigotes may exploit localized immunological niches, such as the granulomatous microenvironment of RNs, where the antimicrobial activity may be impaired, and the immune surveillance decreased, to survive, proliferate, and, possibly, disseminate.

From a therapeutic point of view, the management of CL in immunocompromised patients is challenging. Treatments must be tailored to clinical form, infecting species, and the patient’s factors. Given the emerging resistance and the toxicity associated with pentavalent antimonials, which are still considered the first-line therapy in many endemic countries, we opted for liposomal amphotericin B, which represents, nowadays, a second-line treatment for CL. Several reports have documented its efficacy against *L. major* when administered both intralesionally and intravenously [13,19]. In our case, systemic treatment with liposomal amphotericin B resulted in significant clinical results, supporting the previously reported effectiveness against *L. major*.

## 4. Conclusions

This case presents a rare and intriguing clinico-pathological association between DCL and RNs in a patient with long-standing RA, treated with chronic systemic corticosteroid therapy. The identification of *L. major* DNA within a pre-existing RN represents an exceptional finding, which has been previously reported in the literature in a handful of cases because of relapsing or recurring disease in immunocompromised patients. To our knowledge, our observation of primary DCL in this immunosuppressed patient has not been reported before, and it might lead to further investigative research concerning the possible etiopathogenetic mechanisms underlying it.

This case expands the differential diagnosis of RN, highlighting the importance of considering infections, including leishmaniasis, in the differential diagnosis of atypical clinical presentations, particularly in patients receiving chronic immunosuppression, who come from or travel to endemic regions. The intracellular pathogens may exploit the localized immunological niche represented by the RN of an immunocompromised host to survive and replicate undisturbed. It also underscores the diagnostic value of the clinico-pathological correlation, emphasizing the importance of maintaining a high index of suspicion for opportunistic infections when evaluating immunocompromised patients with exuberant cutaneous lesions, and the importance of integrating the molecular analysis to identify unexpected and rare parasitic infections that can be hidden by concomitant autoimmune diseases, avoiding misdiagnosis, ensuring timely initiation of the most effective treatment, and improving patients outcomes. Dermatologists and dermatopathologists should be aware that leishmaniasis, although considered a neglected tropical zoonosis, may present in unexpected forms also in non-endemic countries, as globalization facilitates travel, and the establishment of new immunomodulatory therapies permits patients a chronic coexistence with autoimmune diseases.

## Data Availability

All the data generated from this study are reported in the manuscript.
